# 6-Sulphated Chondroitins Have a Positive Influence on Axonal Regeneration

**DOI:** 10.1371/journal.pone.0021499

**Published:** 2011-07-01

**Authors:** Rachel Lin, Thomas W. Rosahl, Paul J. Whiting, James W. Fawcett, Jessica C. F. Kwok

**Affiliations:** 1 Department of Clinical Neurosciences, Cambridge Centre for Brain Repair, University of Cambridge, Cambridge, United Kingdom; 2 The Neuroscience Research Centre, Merck, Sharpe and Dohme, Harlow, United Kingdom; The Mental Health Research Institute of Victoria, Australia

## Abstract

Chondroitin sulphate proteoglycans (CSPGs) upregulated in the glial scar inhibit axon regeneration via their sulphated glycosaminoglycans (GAGs). Chondroitin 6-sulphotransferase-1 (C6ST-1) is upregulated after injury leading to an increase in 6-sulphated GAG. In this study, we ask if this increase in 6-sulphated GAG is responsible for the increased inhibition within the glial scar, or whether it represents a partial reversion to the permissive embryonic state dominated by 6-sulphated glycosaminoglycans (GAGs). Using *C6ST*-1 knockout mice (KO), we studied post-injury changes in chondroitin sulphotransferase (CSST) expression and the effect of chondroitin 6-sulphates on both central and peripheral axon regeneration. After CNS injury, wild-type animals (WT) showed an increase in mRNA for *C6ST*-1, *C6ST*-2 and *C4ST*-1, but KO did not upregulate any CSSTs. After PNS injury, while WT upregulated *C6ST*-1, KO showed an upregulation of *C6ST*-2. We examined regeneration of nigrostriatal axons, which demonstrate mild spontaneous axon regeneration in the WT. KO showed many fewer regenerating axons and more axonal retraction than WT. However, in the PNS, repair of the median and ulnar nerves led to similar and normal levels of axon regeneration in both WT and KO. Functional tests on plasticity after the repair also showed no evidence of enhanced plasticity in the KO. Our results suggest that the upregulation of 6-sulphated GAG after injury makes the extracellular matrix more permissive for axon regeneration, and that the balance of different CSs in the microenvironment around the lesion site is an important factor in determining the outcome of nervous system injury.

## Introduction

Following injury to the mammalian central nervous system (CNS), regenerating axons have to overcome several inhibitory challenges in order to regenerate successfully. Infiltration of reactive astrocytes, oligodendrocyte precursors, microglia/macrophages, meningeal cells and vascular endothelial cells into the injured area causes the formation of a glial scar [1]–[4]. The scar tissue limits damage by controlling the inflammation process and preventing secondary damage to the surrounding tissue [5]–[7]. On the other hand, the scar itself is a reservoir of inhibitory molecules which axons are unable to penetrate, particularly due to upregulation of inhibitory chondroitin sulphate proteoglycans (CSPGs) [8]–[9].

Chondroitin sulphate (CS) chains are thought to play an essential role in axonal guidance and regeneration, and are responsible for most of the inhibitory activity of CSPGs [10]–[13]. Because most CSPGs are inhibitory to axon regeneration, chondroitinase ABC has been injected into the damaged CNS to digest CS-GAG chains, in most cases resulting in increased axon regeneration [1], [3], [14]. This has focused attention on the changes in the GAG chains that accompany CNS damage.

CSPGs also play a part in restricting recovery from CNS damage through their presence in perineuronal nets (PNNs). At the end of the critical periods for plasticity, PNNs form around many CNS neurons, playing a key part in the reduction of plasticity. Digestion of CSPGs in the adult CNS reactivates plasticity, allowing improved functional recovery after damage [15]–[17]. It has been demonstrated that enzymatic removal of CS from embryos can disrupt developmental axonal navigation [10], [12].

CS can bear sulphate groups at position 4 and/or position 6 on *N*-acetylgalactosamine (Gal*N*Ac) residues, and position 2 or 3 on glucuronic acid (GlcA) residues. Sulphated CS-glycosaminoglycans (GAGs) are synthesised by a family of chondroitin sulphotransferases (CSSTs) that transfer sulphate from its high energy donor 3′-phosphoadenosine 5′-phosphosulphate to the corresponding carbon on the Gal*N*Ac or GlcA residues in the Golgi apparatus [18]. Mono-sulphation in the C4 or C6 of Gal*N*Ac residue results in the production of CS-A (or C4S) and CS-C (or C6S) respectively, di-sulphation in C2 of GlcA and C6 positions of Gal*N*Ac creates CS-D, while sulphation in both C4 and C6 of Gal*N*Ac produces CS-E [19]. Four families of CSSTs have been identified: (1) chondroitin 4-sulphotransferases (C4STs); (2) chondroitin 6-sulphotransferases (C6STs); (3) *N*-acetylgalactosamine 4-sulphate 6-sulphotransferase and; (4) uronyl 2-sulphotransferase (U2ST). Each of the sulphotransferases is responsible for the sulphation of Gal*N*Ac4S, Gal*N*Ac6S, Gal4S6S, and GlcA2S, respectively on the CS disaccharide (ΔDi) unit [20].

Although CSPGs inhibit axon growth in general, CS chains with different sulphation patterns confer various degrees of inhibition on axon growth, with different studies showing that CS-A and CD-E are inhibitory [21]–[22], while for embryonic axons the disulphated forms are permissive [23]–[24]. It is likely that upregulation of specific sulphation pattern(s) on CS-GAG after injury might be responsible for different levels of growth inhibition. During development there is a progressive shift from predominantly 6-sulphated GAG in embryos to 4-sulphated CS GAG in adults [17], [25]. Following injury there is a large overall increase in GAGs, and three studies have found rather different changes in the GAG-sulphating enzymes and patterns of sulphation. One study into injury-related CSST upregulation in the damaged cortex in rats found *C6ST*-1 (Accession: NM_016803.3) to be upregulated while another study showed increased 6 and 4,6-sulphated GAGs, and a third study of injured spinal cord found an increase in 4-sulphated GAG [21]–[22], [26]. *C6ST*-1 upregulation has been associated with Schwann cell-assisted guidance of axons through the developing and injured environment [27].

The preceding evidence indicates that the pattern of CS-GAG sulphation will have an important influence on the outcome of damage to the nervous system, and it is therefore important to study the role of the different sulphation patterns on regeneration. The *C6ST*-1 knockout mice (*C6ST*-1 KO) are of particular interest for studying the role of CSs in the regeneration response and in plasticity after nervous system injury. Despite C6S being almost undetectable in multiple organs in the *C6ST*-1 KO, the null mutant mice develop normally [28]. Only a decrease in the number of naïve T lymphocytes in the spleen of 5–6-week-old mutant mice was observed. The seemingly normal appearance of such mutants may be accounted for by other enzyme(s) that have *C6ST* activity, such as *C6ST*-2 (Accession: NM_018763) and *N*-acetylgalactosamine 4-sulphate 6-sulphotransferase. Here, we characterised the regenerative ability and the level of plasticity of the nervous system in this animal model. Axon regeneration was examined in both the CNS and the peripheral nervous system (PNS). We examined the level of plasticity in the spinal cord using a model we have developed in which return of skilled paw reaching behaviour is assessed after repair or crossover of the median and ulnar nerves [14]. Finally, regulation of CSSTs upon insults to the CNS and PNS were investigated to see whether other CSSTs are upregulated to compensate for the lack of *C6ST*-1.

## Results

### *C6ST*-1 KO mice lack C6ST-1 mRNA and have reduced 6-sulphated GAGs

**Figure 1:**
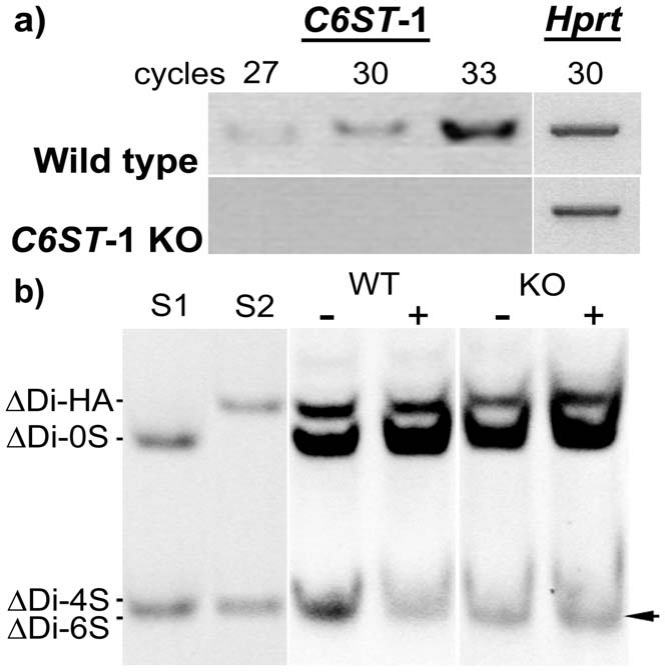
*C6ST*-1 mRNA or 6-sulphated GAGs were not expressed in *C6ST*-1 KO mice. (a) Expression of *C6ST*-1 mRNA after 27, 30, and 33 RT-PCR cycles. *C6ST*-1 mRNA is present in wild-type animals but absent in *C6ST*-1 KOs. The amplification of housekeeping Hprt acts as a positive control. (b) FACE gel showed a decrease of 6-sulphated GAGs in *C6ST*-1 KO animals compared to WT mice (lanes marked with “−”). Removal of 6-suphated moieties, by treatment with chondroitinase ABC and chondro-6-sulfatase (lanes marked with “+”) before the conjugation with 2-aminoacridone, showed that a sharp decrease in band intensity at ΔDi-4S or ΔDi-6S position in WT samples. However, there was no change in intensity in KO samples, suggesting the lack of ΔDi-6S. Arrow points to 6-sulphated products. **S1** and **S2** are the ΔDi standards.

Expression of *C6ST*-1 mRNA was determined by semi-quantitative RT-PCR with 27, 30, and 33 cycles of amplifications. The results demonstrated that while *C6ST*-1 mRNA is present in WT and showed increasing band intensity with increasing cycle numbers, *C6ST*-1 was not expressed in the *C6ST*-1 KO. No band was observed in all cycle numbers examined (Figure 1a). FACE analysis was carried out to determine the disaccharide (ΔDi) composition in both the *C6ST*-1 KO and WT brains (Figure 1b). Compared with controls, the KO showed a decrease in the overall size of the combined band for 6-sulphated and 4-sulphated ΔDi (lanes marked with “-” in figure 1b). The proportion of the two disaccharides was determined by using chondro-6-sulphatase to remove the 6-sulphated ΔDi. While the band intensity of the ΔDi-4S/ΔDi-6S decreased drastically after 6-sulphatase treatment in the WT samples, there was no change in intensity in the KO samples (lanes marked with “+” in figure 1b), suggesting that in the *C6ST*-1 KO, there is very little 6-sulphated GAG.

### Diminished regeneration of nigrostriatal TH-positive axons in *C6ST*-1 KO

**Figure 2:**
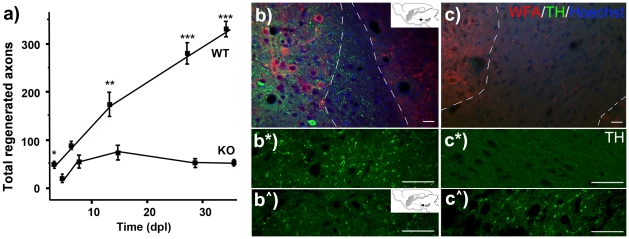
Nigrostriatal TH-positive axons regeneration in *C6ST*-1 KO mice. Axons showed significant recovery in WT animals but not in *C6ST*-1 KO animals after axotomy. (a) A graph showing the number of nigrostriatal axons regenerated after axotomy. Total number of regenerating axons was greater in the WT than in *C6ST*-1 KOs. During the course of the experiment, total regenerated axons in WT climbed to over 300, while the number of regenerating axons consistently remained well below 100 over the experimental period. (Day 4: *p* = 0.021, *; Day 7: *p = *0.083; Day 14: *p = *0.007, **; Day 28: *p*<0.001, ***; Day 35: *p*<0.001, ***). (b, c) Confocal images of brain sections showing regenerating axons 28 days post nigrostriatal axotomy, visualised with TH staining (green). WFA (red), Wisteria floribunda agglutinin, labels the CSPGs in the PNNs without injury. The sections were counter-stained with Hoechst's staining. (b, b* and b∧) Wild-type, (c, c* and c∧) *C6ST*-1 KO. (b* and c*) High magnification images of the TH staining in the injured site (corresponds to the black rectangular box in schematic diagram in b). (b∧ and c∧) High magnification images of the TH staining in the nigrostriatal tract distant from the injury sites (corresponds to the black rectangular box in schematic diagram in bˆ). Scale bar, 25 µm.

To test the ability of WT and *C6ST*-1 KO to support axon regeneration, we counted the number of TH-positive axons regenerated across injuries to the nigrostriatal tract. These axons have a high regenerative ability, and in mice show some spontaneous regeneration [29]–[30]. The nigrostriatal tract is therefore an appropriate pathway to test whether the CNS environment becomes more or less permissive to axon regeneration as a result of changes in CS-GAG sulphation. The total number of regenerating axons in the *C6ST*-1 KO was much less than that of the WTs (Figure 2a). In WT, the number of axons regenerating across the injury increased steadily in the course of 35 days after injury, but the number of axons in the KO remained low during the same period. Moreover, there was considerable retraction of TH-positive axons from the lesion site in the *C6ST*-1 KO (Figure 2). Differences in the number of regenerating axons were significant in all the time points examined except at day 7 (Day 4: *p* = 0.021, Day 7: *p = *0.083, Day 14: *p = *0.007, Day 28: *p*<0.001, Day 35: *p*<0.001).

### In the PNS: C6ST-1 KO showed similar regenerative ability to the wildtype

**Figure 3:**
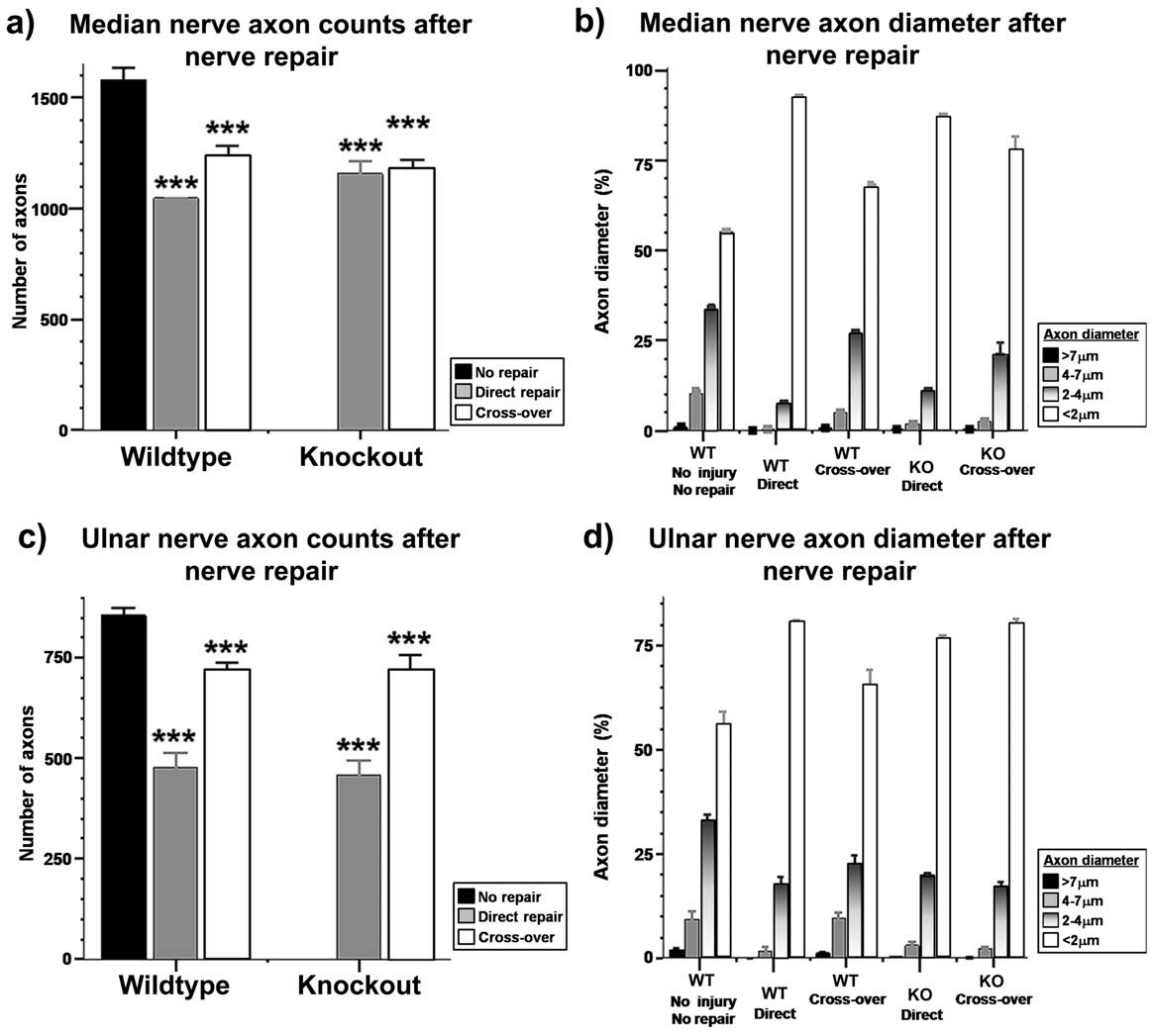
Changes of axon number and diameter between *C6ST*-1 KO and normal mice after PNS injury. (a) Number of median nerve axons is just above 1500 in uninjured animals. After peripheral nerve injury (direct or cross-over), the number of axons decrease significantly compared to non-injured animals in all genotypes examined (*p*<0.001, ***). No significant difference can be found between the injured groups. (b) The distribution of axon diameter altered after injury. Proportion of larger diameter axons (>4 µm) diminished after nerve transection and repair. (c) and (d) Ulnar nerve axon numbers are different between *C6ST*-1 KO and normal mice after nerve injury and repair. (c) Number of median nerve axons dropped from 800 to as low as 500 in animals that received direct repair. After peripheral nerve injury (direct or cross-over), the number of axons decrease significantly compared to non-injured animals in all genotypes examined (*p*<0.001, ***). In each genotype, animals that received cross-over repair had significantly more regenerated axons (*p*<0.001) compared to those that received direct repair. (d) The distribution of axon diameter altered after injury. Proportion of larger diameter axons (>4 µm) diminished after nerve transection and repair.

To test axon regeneration in the PNS the median and ulnar nerves were cut then rejoined with either a direct or cross-over repair. Resin embedded nerve sections stained with toludine blue were examined using light microscopy and the number of regenerated myelinated axons in the distal segment of the nerve was quantified. The total number of median and ulnar nerve axons after 40 weeks of regeneration (both WT and C6ST-1 KO) was less than that in non-injured animals (*p*<0.001). No significant difference was found between the injured groups. The distribution of axon diameter also altered after injury (Figure 3b). The proportion of larger diameter axons (>4 µm) diminished after nerve transection and repair, while the proportion of smaller diameter axons, especially those with a diameter of less than 2 µm increased markedly (Figure 3 b, d).

#### Skilled paw-reaching tasks: dexterity and grip strength

**Figure 4:**
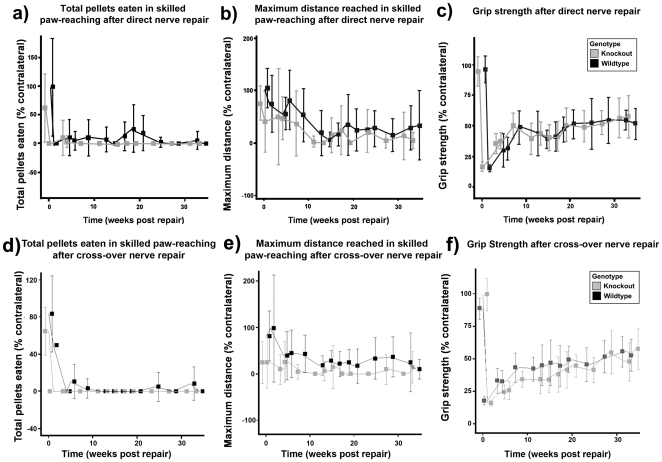
Functional assessment after direct and cross-over peripheral nerve transection and repair. (a, d) Total pellets eaten during skilled paw-reaching task. After median and ulnar nerve repair (both direct and cross-over), pellets eaten dropped significantly (*p*<0.001) on the operated side and stayed down close to such values for the entirety of the experiment. (b, e) Maximum distance reached during skilled paw-reaching task. Distance reached by the operated side compare to normal side in both groups of animals dropped steadily following surgery, however, the decrease in distance was not as dramatic as pellets eaten immediately following nerve repair. (c, f) There is a significant decrease in grip strength immediately after surgery followed by steady increase over the experimental period. No difference can be seen between wild-types and *C6ST*-1 KOs.

Animals with direct (median to median, ulnar to ulnar) or crossover repairs were assessed for recovery of forelimb function as a measure of spinal cord plasticity, using methods established in our previous studies [14], [31]. Functional recovery in the PNS was assessed by skilled paw-reaching and grip strength tasks. Animals were pre-trained for three weeks, then the baseline (week 0) was established just before nerve transection and repair. Behavioural tests started three weeks after peripheral nerve repair. In the direct repair group, there were no significant differences in recovery between WT and *C6ST*-1 KO. Total pellets eaten during the skilled paw-reaching task after direct median and ulnar nerve repair dropped significantly on the operated side and stayed close to such values for the entire period of the experiment (Fig. 4a). Distance reached by the operated side dropped in both groups of animals and remained diminished (Figure 4b). There was also a significant decrease in grip strength immediately after surgery followed by a steady increase over the experimental period (Fig. 4c).

Similar to the direct repair model, following cross-over peripheral nerve injury and repair, animals showed a significant decrease in both total pellets eaten and forelimb grip strength in both the WT and *C6ST*-1 KO (Figure 4, d to f). The maximum distance reached by the two groups of animals remained consistent before and after surgery.

#### Muscle weight

**Figure 5:**
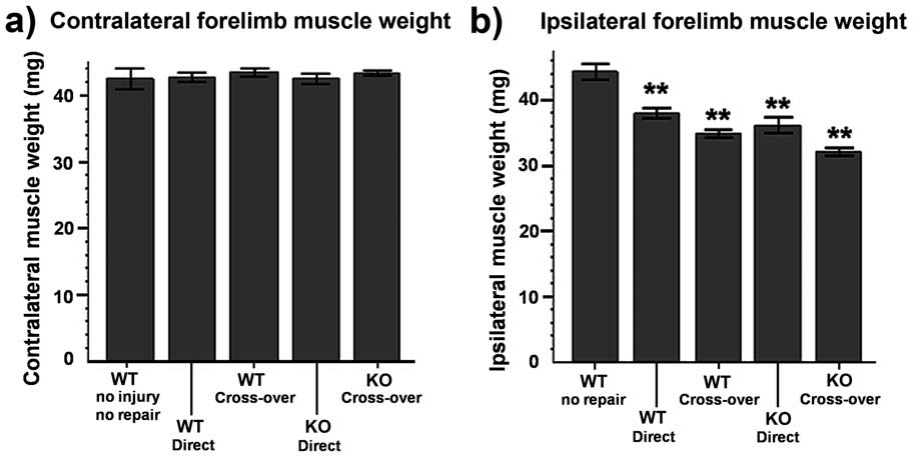
Forelimb muscle weight decreased after median and ulnar nerve injury and repair. (a) No significant difference was observed between operated but non-repaired and non-operated contralateral flexor carpi radiali weights between all groups. (b) Muscle weights of non-repaired animals were significantly higher than all animals that received a nerve injury (*p*<0.01, **). No difference in muscle weight exists between the repaired groups except WT direct repair and *C6ST*-1 KO cross-over repaired animals (*p*<0.01, **).

After sacrificing the animals, flexor carpi radialis (FCR) were dissected out and weighed. The weight of the muscles gives an indication of the degree of re-innervation [14]. The weights of the FCR muscle were the same between genotypes examined (wild-type and *C6ST*-1 KO) (Figure 5). The muscle weight of sham operated (no nerve lesion) animals was significantly higher than all groups which received nerve repairs (direct and cross-over repair) (Figure 5b) indicating that muscle re-innervation was incomplete. The muscle weights recorded demonstrated mild muscle atrophy, typical of re-innervation of the muscle after nerve repair. No difference in muscle weight existed between the repaired groups except WT direct repair and C6ST-1 KO cross-over repaired animals (*p*<0.01, **).

### Changes in sulfotransferase expression after injury in the PNS and CNS

**Figure 6:**
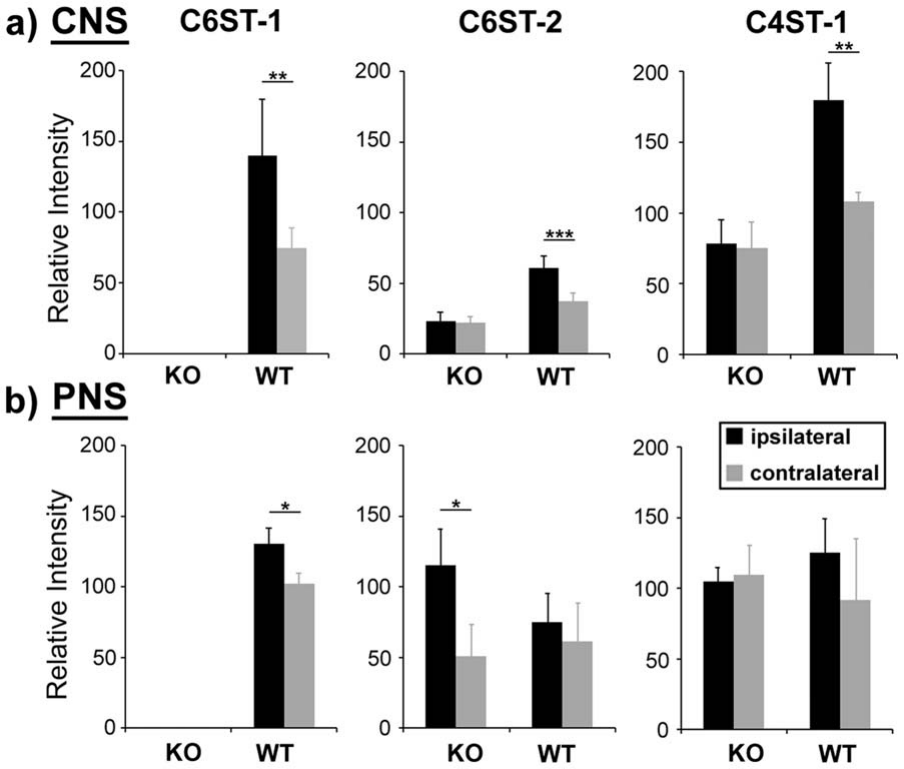
Regulation of CSST mRNAs after nervous system injury. The mRNA of 6-sulphate-related sulphotransferases was upregulated in the (b) PNS after injury but not in the (a) CNS . a) *C6ST*-1 mRNA cannot be detected in *C6ST*-1 KO animals with cortical stab injury. After the injury, the level of *C6ST*-1 (*p* = 0.001, **), *C6ST*-2 (*p*<0.001, ***), and *C4ST*-1 (*p* = 0.006, **) mRNAs were upregulated in normal wild-type animals. No regulation mRNA levels was observed in *C6ST*-1 KO animals after injury. b) *C6ST*-1 mRNA cannot be detected in *C6ST*-1 KO animals after PNS injury. After nerve crush injury, the level of *C6ST*-1 mRNA was upregulated (*p* = 0.011, *) in WTs. No regulation of *C6ST*-2 was observed in WT animals, but a significant upregulation was seen in *C6ST*-1 KO mice (*p* = 0.036, *). Five separate tissues were processed for CSST mRNA regulation analysis.

We investigated the expression of the various CSSTs 7 days after CNS and PNS injury in both WT and KO. mRNA extracted from the uninjured contralateral sides were used as control. A 7 day time-point was chosen because it has been shown to be the peak of expression of inhibitory CSPGs and CSST upregulation [26], [32]. The expression of CSST mRNAs were quantified and normalised against the GAPDH results before and after injury using the semi-quantitative PCR approach. *C6ST*-1 mRNA was not detected in either the CNS or PNS of the *C6ST*-1 KO (Figure 6). After CNS injury, the mRNA levels of *C6ST*-1 (*p* = 0.001, **), *C6ST*-2 (*p*<0.001, ***), and *C4ST*-1 (*p* = 0.006, **) were upregulated in the WT (Figure 6a). In the *C6ST*-1 KO, there was no change in the mRNA expression of any of the CSSTs investigated (Figure 6a).

The mRNA expression of the same CSSTs before and after peripheral nerve crush was also quantified (Figure 6b). As in the CNS, the level of *C6ST*-1 expression was increased in WT (*p* = 0.011, *) but the increases in *C6ST*-2 and *C4ST*-1 that were seen in the CNS were not present after the PNS lesions. In the *C6ST*-1 KO expression of C6ST-2 doubled after PNS injury (*p* = 0.036, *). No regulation of the other CSSTs was observed (figure S1).

## Discussion

Structural diversity of CS-GAG chains, resulting from sulphation by different CSSTs, plays a crucial role in various biological functions of CSPGs. *C6ST*-1, the enzyme knocked out in this study, transfers sulphate to the C6 position of the Gal*N*Ac residue of CS during the last step of its synthesis. 6-sulphated CS-C is the predominant form of CS during embryogenesis, being replaced by 4-sulphated CS-A in adulthood [25]. *C6ST*-1 is upregulated after CNS injury and its product CS-C is also selectively enriched [26]. Thus, the increase in *C6ST*-1 and 6-sulphated products may have important implications for axon regrowth and plasticity in the CNS, which are influenced by CSPGs. However, there have been conflicting findings on whether CS-C or CS-A is the more inhibitory CS to axon regeneration [21]-[22], [33]. Depending on the relative inhibitory potential of these two forms of CS, the relative upregulation of 6-sulphated CS in the injured CNS could be interpreted as 1) leading to a more inhibitory barrier to the regeneration of CNS axons, or 2) as an attempt to return the CNS to a more permissive developmental state. The *C6ST*-1 deficient mice provide a tool to resolve this issue. We find that in the absence of the *C6ST*-1 gene, the damaged CNS shows even less axonal regeneration than normal, and possesses a low level of plasticity, suggesting that 6-sulphated GAG (a product from both *C6ST-1* and *-2*) is permissive rather than inhibitory.

### WT and *C6ST*-1 KO differ in their pattern of CSST response to injury

We examined the changes in CSST expression in the injured brain and median nerve, using a semi-quantitative PCR approach. In wild-type animals both the *C6ST*s and *C4ST*-1 mRNAs were significantly upregulated 7 days after cortical stab injury, similarly to previous studies, which have reported variously increases in 4, 6 and 4,6 sulphated GAGs [21]–[22], [26]. Surprisingly, in the *C6ST*-1 KO, no significant upregulation of the other CSSTs was observed after injury (Figure 6). Peripheral nerve injury, on the other hand, showed a different pattern of CSST mRNA regulation. After median and ulnar nerve crushes, the WT animals showed an upregulation of *C6ST*-1 expression. However, unlike the CNS, *C4ST*-1 expression did not increase. In *C6ST*-1 KO, the expression of *C6ST*-2 was strongly upregulated after nerve injury (Figure 6b) whereas the expression of other CSSTs was unchanged as in WTs (figure S1). Expression levels of CSSTs and their respective products are found to be closely connected [26], so the changes that we have seen in CSST expression will be reflected in changes in GAG sulphation. This means that in the knockout animals there will be an increase in 6-sulphated GAG after PNS injuries due to upregulation of *C6ST*-2, but this increase will not occur after cortical injury.

### Absence of *C6ST*-1 expression is deleterious to axon and regeneration in the CNS but not the PNS

We examined the regenerative ability of cut CNS axons in the nigrostriatal pathway. The axons of this pathway have a high regenerative potential and, unusually for CNS axons, show a small amount of spontaneous regeneration after axotomy in mice [29]–[30]. This is therefore a suitable pathway in which to look for either an increase or a decrease in axon regeneration. When we examined the number of regenerated axons in *C6ST*-1 KO, we found essentially no regeneration (Figure 2a). In contrast, there were regenerative sprouts in WT animals at all time points investigated, with a maximum of 300 axons 35 days after axotomy. The results suggest that the presence of 6-sulphated chondroitin species in the CNS after injury promotes axonal regeneration.

We also examined axon regeneration in peripheral nerves after cutting and then surgically repairing the median and ulnar nerves. There was substantial axon regeneration, after which as usual there was a greater proportion of small diameter axons and less larger axons compared to uninjured controls [34]. However, despite the deleterious effect on CNS regeneration of the *C6ST*-1 KO, there was no difference between WT and *C6ST*-1 KO in the pattern of peripheral nerve regeneration. The success of peripheral nerve regeneration in both genotypes can also be assessed by weighing re-innervated muscles, because re-innervation prevents muscle atrophy. We found in all our animals that the weight of the flexor carpi radialis muscles was consistent with partial re-innervation by regenerated axons, consistent with the approximately 70% recovery of axon numbers in the distal part of the nerves, but there were no differences between the *C6ST*-1 KO and WT.

Overall our axon regeneration results are consistent with 6-sulphated GAG being more permissive to regeneration than 4-sulphated GAG. In the PNS WT increased the expression of *C6ST-1* after injury, and the KO showed a compensatory increase in *C6ST*-2 expression. This suggests that the successful regeneration of PNS axons in these animals is aided by the upregulation of 6-sulphated GAG but not other GAG species. This is also consistent with our findings in the CNS of the KO which lacks this upregulation of 6-sulphated GAG production after injury, and in which axon regeneration is reduced. The other change in the knockouts is the lack of upregulation of *C4ST-1* after injury, but the expression level of *C4ST-1* is still relatively high in both the PNS and CNS. When the results from the PNS and CNS are considered together, it is more likely that the reduced CNS regeneration is due to the very low level of 6-sulphated GAG. Indeed, a recent study by Wang *et al.* showed that chondroitin 4-sulphate, but not chondroitin 6-sulphate, negatively regulates axonal growth *in vitro* and another study implicated 4,6 sulphated CS [21]–[22]. The upregulation of *C6ST*-1 and therefore 6-sulphated chondroitin species, therefore represents a partial return to the GAG composition that is present during development, and appears to make the CNS environment more permissive to axon regeneration.

Upregulation of *C6ST*-1 gene expression has been suggested to be correlated with Schwann cell mobility after injury. Down-regulation occurs in Schwann cells when they differentiate to the myelinating phenotype [27]. It has been hypothesized that the migrating Schwann cells upregulated *C6ST* genes to produce 6-sulphated chondroitin moieties to counteract cell adhesiveness to help the cells migrate and guide axons through the environment.

### Absence of *C6ST*-1 does not increase spinal cord plasticity

An important function of CSPGs in the CNS is the regulation of the level of plasticity, through their participation in perineuronal nets (PNNs). PNNs are condensed extracellular matrix structures that form around the cell body and dendrites of many CNS neurons at the time when plasticity critical periods terminate. They contain a mixture of CSPGs, hyaluronan, link proteins and tenascin-R [35]. The pattern of sulphation of the CSPGs in PNNs differs from that of the diffuse matrix found throughout the CNS [36]. Digestion of these structures with chondroitinase ABC reactivates CNS plasticity, and prevention of their formation in animals deficient in cartilage link protein-1 prolongs juvenile levels of plasticity into adulthood [17]. We developed an assay for the level of plasticity in the spinal cord, which we have used to assess plasticity in the *C6ST*-1 KO. After direct repair or crossover repair of the severed median and ulnar nerves, many axons regenerate to peripheral targets. However, due to a lack of guidance mechanisms, they mostly connect to the wrong muscle or sensory structure. The result is very poor skilled paw function, as demonstrated by animals in the current experiment. Injection of chondroitinase ABC into the cord reactivates plasticity and allows recovery of skilled paw reaching function [14]. If the lack of 6-sulphated CS in PNNs leads to an increase in CNS plasticity, we would therefore expect that *C6ST*-1 KO would recover paw reaching after nerve repair to a greater extent than control animals. However, the knockout animals showed little recovery over the entire experimental period indicating a low level of spinal cord plasticity. In terms of grip strength recovery there was also no significant difference between control and KO animals. We can conclude that the lack of *C6ST*-1 does not increase CNS plasticity.

In conclusion, the pattern of axon regeneration and functional recovery in the *C6ST*-1 KO suggest that 6-sulphated CS species has a positive effect on axon regeneration in both PNS and CNS. The upregulation of C6ST-1 and 6-sulphated GAG after injury is therefore a partial return to the more permissive environment of the embryonic nervous system, but is not sufficient to secure regeneration of CNS axons. The absence of 6-sulphated GAG in perineuronal nets must change the binding properties of these structures, but it does not lead to a greater level of plasticity in the adult CNS.

## Materials and Methods

### Surgical Procedures and Tissue Processing

#### Animal subjects and general procedures

All animal experiments were carried out in accordance with the UK Animals (Scientific Procedure) Act of 1986. The experiments were approved by the Cambridge local ethical committee and the UK Home Office under project licence 80/1557. All procedures used were described in section 19b. Adult *C6ST*-1 KO and wild-type littermates weighing 20–30 g were used in all experiments.

The *C6ST*-1 KO was generated by Deltagen, Inc., San Mateo, CA (T1585) by gene targeting and licensed mice were provided by Merck & Co. The animals were bred as homozygotes, and the absence of the *C6ST*-1 gene was confirmed by reverse-transcription polymerase chain reaction (RT-PCR).

#### Methods of anaesthesia

The mice were anaesthetised with 3.5% isoflurane in carrier gas (oxygen, 2 L/min) and transferred to a stereotaxic frame. Anaesthesia was maintained thereafter with isoflurane (1.0–2.0%) in carrier (oxygen, 0.6 L/min) with inhalation analgesic (nitrous oxide, 0.4 L/min)

#### Medial forebrain bundle (nigrostriatal tract) lesion

A Scouten wire knife (Kopf Instrument) was used to transect the nigrostriatal tract. A dental drill was used to expose a hole over the skull at AP -1.1 mm and ML -1.5 mm lateral relative to the bregma. The dura was referenced, gently pierced and the Scouten knife lowered through the hole to −4.9 mm ventral below the dura. The blade was extruded 1.5 mm, raised 1.5 mm, and retracted before extruded again and lowered 1.5 mm back to the original starting point. The blade was retracted and removed from the brain.

Animals were terminally anaesthetised with sodium pentobarbitone (2 ml/kg, Euthatal, Rhône Merieux) at 4, 7, 14, 28, and 35 days post lesion (*n* = 5 per time point per group) followed by perfusion through the heart with 20 ml phosphate-buffered saline prewash (pH 7.4) and the 20 ml of 4% paraformaldehyde (PFA, pH 7.4). The brain was removed and post-fixed for 24 h at 4°C then transferred to 30% sucrose (4°C) until cryoprotected. The right hemisphere of each brain was sliced into 40 µm sagittal sections using a sledge microtome, and stored in Trizma (Sigma)-buffered saline (TBS) with 0.05% sodium azide at 4°C.

#### Cortical stab injury

A dental drill was used to remove a section of the skull starting from 1 mm lateral to bregma and extending rostrally to lambda. A sterile blade (size 11) was lowered (1.8 mm ventral relative to dura) into the cortex and drawn along the length of the exposed brain, parallel to the midline. Animals were terminally anaesthetised at 7 days post lesion (*n* = 10 per genotype), a ±1 mm area surrounding the stab and the corresponding area in the contralateral side were dissected out and frozen for RNA extraction or ΔDi composition analysis.

#### Peripheral nerve repair

The median and ulnar nerves were injured and repaired in the left forelimb only, leaving the right to serve as an uninjured control. During the surgery the mouse lay supine with the left forelimb fully extended by a ligature around the wrist. A 1 cm incision was made on the ventral surface of the limb from the elbow towards the clavicle. The skin was retracted, connective tissue cleared, and epineurium was removed with a pair of fine forceps to expose the nerves. Both nerves were injured about 3 mm proximal to the elbow joint. A crush lesion was induced three times for 20 seconds each using a pair of serrated forceps (*n* = 5 per genotype). For direct repair, median nerve was cut and then stitched back using one epineurial stitch with 10–0 sutures (*n* = 10 per genotype). The procedure was then repeated for the ulnar nerve. For cross repair, both median and ulnar nerves were cut and then the proximal stump of the median nerve was sutured to the distal stump of the ulnar nerve and vice versa (*n* = 10 per genotype). The skin of the forelimb was stapled using Michel suture clips (FST).

Animals were terminally anaesthetised at 7 days post crush lesion and both median and ulnar nerves dissected out and frozen immediately for RNA extraction. At end of behaviour study (7 months) the nerve repaired animals were terminally anaesthetised, flexor carpi radialis removed and weighed while the median and ulnar nerves were dissected out and fixed in 4% PFA overnight.

### Behavioural Testing

#### Staircase Test

The staircase test is designed to evaluate skilled paw reaching and grasping in the forelimb [31], [37]–[38]. One sucrose pellet (NOYES Precision Pellets) was placed onto each step of two staircases located on either side of a central plinth. Animals were placed in the holding box with access to the plinth and staircase and given 20 min habituation period each day in the staircase (Model 80301, Campden Instruments) during the initial week. The mice were given one further week to train following by another pre-training week with only the lower 5 steps (wells) baited with pellets (the upper 3 wells were devoid of pellets since animals were able to retrieve these pellets by their tongue). At the end of the three week training period, the mice were tested prior to surgery (baseline) and twice a week following peripheral nerve repair. Pellets eaten or displaced by the mouse at the end of each testing sessions were recorded. While number of pellets eaten indicates success grasping and retrieval of the pellet, pellet displacement gives an indication of how far the mouse can reach.

#### Grip Strength

Forelimb grip strength was measured using a modified lateralised grip strength meter (model GSM1054; Linton Instrumentation). The apparatus was modified to give two coaxially arranged grip rods connected to two separate strain gauges which were able to quantify the applied force to each rod. On each trial, the mouse was held by the tail and lowered to the bars until it griped the two bars, one with each forepaw. The mouse was then pulled by the base of the tail away from the bars until the grip loosened. The applied forces at which the mouse released the rods were recorded. The mice were tested prior to surgery (baseline) and then once a week after nerve repair. Each test session consisted of three trials.

### Histology

#### Free-floating sections

Free-floating PFA-fixed brain sections were quenched (10% methanol and 3% H_2_O_2_ in distilled water) for 5 min, washed with TBS (pH 7.4) then blocked in 3% normal goat serum (NGS, Dako) with TBS containing 0.2% Triton X-100 (Sigma, TXTBS) for 1 hr, room temperature (RT). Sections were incubated overnight with the primary antibody in TXTBS with 1% NGS at 4°C. Antibody to tyrosine hydroxylase (TH, rabbit polyclonal, 1∶4000; Jacques Boy Institute) was used to confirm transacted axons and to help identify regenerating axons. After washing in TBS, sections were incubated in secondary antibody (biotinylated anti-rabbit immunoglobulins, 1∶200 ; Dako or anti-rabbit Alexa 488 nm antibody, 1∶500; Molecular Probes, Invitrogen) for 3 hr (RT) in TXTBS with 1% NGS, and then washed with TBS. Finally, sections were counterstained with Hoechst nucleic stain. Sections were washed in TNS, mounted onto 1% gelatine-coated slides, air-dried and cover-slipped with Fluro-Save mounting medium (CalBiochem).

#### Resin embedded sections

Most distal 7 mm median and ulnar nerve segments from peripheral nerve repair animals were fixed in 4% PFA before being fixed in 2.5% glutaraldehyde and then transferred to 0.1M sodium phosphate buffer. The nerve segments were immersed in 2% osmium tetroxide at 4°C overnight, dehydrated through sequential ethanol solutions (70%, 95% and 100%), immersed in propylenoxide, followed by propyleneoxide-TAAB resin (1∶1 solution), and embedded in TAAB resin and allowed to polymerise overnight at 60°C. One micron thick transverse sections were cut from hardened resin blocks with a microtome (Leica RM2065) and stained using toludine blue and Borax (di-sodium tetraborate) and examined using light microscopy.

### Quantifying regenerating axons

Regenerating TH-positive axons were quantified using a light microscope under 40x objective. Axons which have crossed the site of injury at 4, 7, 14, 28, and 35 days were counted and grouped according to the distance of regeneration (<1 mm, 1–2 mm, 2–3 mm, >3 mm). Axons from n≥3 animals were counted per time point.

### RNA extraction

Total RNA was isolated from cortical tissue surrounding the cortical stab injury, contralateral control tissue, injured and normal median nerves. The RNA was extracted following the TRIzol isolation method according to the manufacturer's protocol (Life Technologies). An additional chloroform extraction step was performed to ensure purity of the RNA. First strand synthesis was performed following protocol for SuperScript III First-Strand Synthesis System (Invitrogen).

#### Semi-quantitative reverse-transcription polymerase chain reaction (RT-PCR)

PCR was performed on the determining the expression of different CSSTs using 50 µM of each deoxynucleotide, 200 µM of each forward and reverse primers, 2 U of Taq DNA polymerase, and specific concentration of MgCl_2_ for each primer pair (Table 1) in each reaction mixture. The mixture was incubated in a thermal cycler at 94°C for 5 min, 94°C for 30 sec, specific melting temperature (Table 1) for 30 sec, 58°C for 30 sec and 72°C for 1 min. Each primer pair reaction was amplified in triplicate with three different cycle numbers. Glyceraldehyde-3-phosphate dehydrogenase (GADPH) or hypoxanthine-guanine phosphoribosyltransferase (Hprt) were used as housekeeping genes and a PCR without Taq DNA polymerase or without cDNA template was also performed to detect any non-specific PCR reaction. The PCR products were electrophoresed and analysed after the PCR with a 1.5% agarose gel. Specific cycle numbers used for semi-quantitative purposes were determined in an initial study; the saturation cycle was used as the maximum cycle number. Final data analysis was based on results obtained from 5 separate samples.

**Table 1:** CSSTs primer sequences and PCR settings.

	Primer Sequence	MgCl_2_ (mM)	Melting Temp (°C)	Cycle Numbers	Product size (bp)
C6ST-1	5′ TTCGTKGGSGAGTTCTTCAAC 3′5′ CTCATAGCGCACCARCATGT 3′	1.0	60	27, 30, 33	674
C6ST-2	5′ TTCAACCAGCACCCGGACG 3′5′ GCACAGAGAAATCACAGCGGAAG 3′	1.5	55	33, 36, 39	142
C4ST-1	5′GAAGCACCTGGTGGTGGATG 3′5′GTAGTTCGGGTGGACTTTGCATAG 3′	1.5	55	24, 27, 30	611
C4ST-2	5′-CACCTCACCTTCAACAAGTTC-3′ 5′-ACTCTTCGTTCTCCAGCTC-3′	1.5	55	24, 27, 30	160
C4ST-3	5′ TCCTATATTGCGGGACAGATTC 3′5′ TGCAGGAGACAGGGGATG 3′	2.0	55	36, 39, 42	148
U2ST	5′ GACCATGGACCACCTCCTAGTAAG 3′5′ GGTGCGGATCATGTGATTCTGT 3′	3.0	55	24, 27, 30	384
GAPDH	5′ TTCCAGTATGACTCTACCC 3′5′ ATGGACTGTGGTCATGAGCCC 3′	1.5	59	21, 24, 27	398
Hprt	5′- AGCTACTGTAATGATCAGTCA ACG -3′ 5′- AGAGGTCCTTTTCACCAGCA-3′	1.5	59	24, 27, 30	200

### Disaccharide composition analysis

#### Glycosaminoglycan purification

A ±1 mm area surrounding the injured cortex was collected and frozen on dry ice for storage. Acetone powder of the sample was prepared by homogenising the samples with chilled acetone and drying by desiccation at 4°C. Acetone powder was suspended in pronase buffer (0.1 M Tris-Ac, 10 mM calcium acetate, pH 7.8) and proteins were digested with pronase (protease from *Streptomyces griseus*, 1∶50, Roche) for overnight at 37°C. Samples were centrifuged at 15,000 rpm for 15 min, the supernatant was collected and cooled on ice. 100% trichloroacetic acid (TCA) was added to the sample (5% final concentration) and incubated on ice for 1 hr, then the supernatant was collected followed by centrifugation (10,000 rpm, 30 min, 4°C). The washing process was repeated and supernatants pooled. Pooled supernatant was washed 5 times with diethyl ether (BDH) and residual ether was allowed to evaporate at 37°C. GAG in the ether-free solution (pH 7.0–7.5) was precipitated with sodium acetate (5% final concentration) and ice-cold ethanol (75% final concentration), and kept at 4°C overnight. Precipitated GAG was collected by centrifugation (3000 rpm, 15 min) and then dried at 4°C. This whole GAG preparation was redissolved in water.

The GAG was treated with 100 mU ChABC and 100 mU chondro-4-sulfatase in 100 mM NH_4_Ac buffer, pH 8.0. The reaction mixtures were incubated at 37°C for at least 16 hs. The reaction was stopped by the addition of 100% ethanol which can also precipitate and recovered the core proteins (4°C, 16 hr). The precipitated materials were then pelleted by centrifugation. The supernatant containing the disaccharides was collected and dried.

#### Analysis of glycosaminoglycans using fluorophore-assisted carbohydrate electrophoresis (FACE)

Procedures for 2-aminoacridone-derivatization of the disaccharides were modified from [39]–[40]. Dried disaccharide preparations were derivatised with 12.5 mM 2-aminoacridone in the presence of 10 µl of 1.25 M sodium cyanoborohydride. Standard disaccharides, including Δdi-HA, Δdi-0S, Δdi-4S, Δdi-6S, were also derivatisation with the 2-aminoacridone using the same method. After the tagging, 10 µl of 30% glycerol was added to the samples and the tagged disaccharides were kept at −20°C.

To analyse the disaccharides, the labelled sample and standard disaccharides were electrophoresed in a 30% polyacrylamide gel in 0.1 M Tris-borate buffers, pH 8.3. Image of the gel was taken by illuminating the gel with 365 nm using the Molecular Imager ChemiDoc XRS System (BioRad). Documentation of the results was done using software “Quantity One” (BioRad).

### Statistical Analysis

All statistical tests were carried out with SPSS (Version 11.0.1). Statistical significance was evaluated for the behavioural tests and CSSTs level with ANOVA. *Post hoc* comparisons were made using the Bonferroni test.

## Supporting Information

Figure S1 **Regulation of CSST mRNAs after nervous system injury.** The expression of other CSSTs mRNA was determined. No regulation of C4ST-2, C4ST-3 and U2ST can be observed in both the a) CNS and b) PNS in WT and KO animals.
(PDF)
