# Supplementary material for: 6-Sulphated Chondroitins Have a Positive Influence on Axonal Regeneration
Source: PLoS One. 2011 Jul 1;6(7):e21499. doi: 10.1371/journal.pone.0021499 (PMC3128591; doi:10.1371/journal.pone.0021499)
Supplement: Figure S1 — Regulation of CSST mRNAs after nervous system injury. The expression of other CSSTs mRNA was determined. No regulation of C4ST-2, C4ST-3 and U2ST can be observed in both the a) CNS and b) PNS in WT and KO animals. (PDF) [file pone.0021499.s001.pdf]

Figure S1

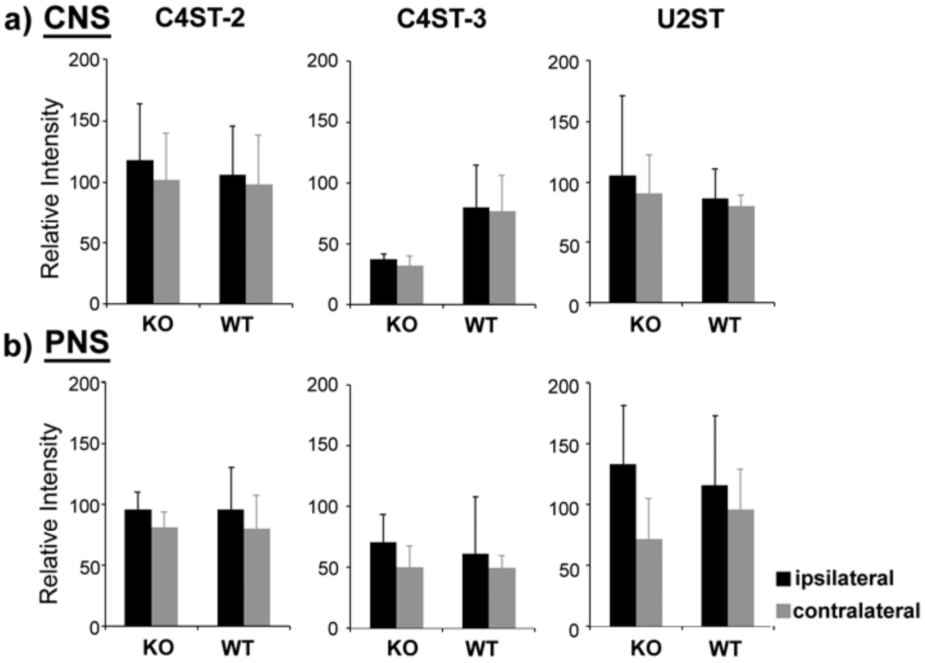

Figure S1. **Regulation of CSST mRNAs after nervous system injury.** The expression of other CSSTs mRNA was determined. No regulation of C4ST-2, C4ST-3, and U2ST can be observed in both the a) CNS and b) PNS in WT and KO animals.
